# Electrocatalytic galvanic cells: a paradigm toward spontaneous chemical synthesis and electricity generation

**DOI:** 10.1093/nsr/nwag396

**Published:** 2026-06-27

**Authors:** Fanhao Kong, Zhong-Shuai Wu

**Affiliations:** State Key Laboratory of Catalysis, Dalian Institute of Chemical Physics, Chinese Academy of Sciences, China; State Key Laboratory of Catalysis, Dalian Institute of Chemical Physics, Chinese Academy of Sciences, China; University of Chinese Academy of Sciences, China

## Abstract

Electrocatalytic galvanic cells redefine electrochemical energy conversion by enabling the spontaneous co-production of chemicals and electricity from renewable and waste feedstocks.

Electrocatalytic galvanic cells (EGCs) represent a new-generation electrochemical technology developed from green and efficient paired electrolysis. In contrast to traditional galvanic cells, where electrical energy originates from redox reactions intrinsic to the electrode materials, EGCs decouple energy from external molecular reactants by employing electrodes that serve as efficient catalysts capable of surface electrocatalytic reactions. Specifically, the anode (negative electrode) is designed to catalyze thermodynamically favorable low-potential oxidation reactions, whereas the cathode (positive electrode) drives high-potential reduction reactions. The intrinsic potential difference between these paired reactions provides the electromotive force to drive spontaneous electron flow through an external circuit, yielding net electricity output without external electricity input. EGCs thus inherit the core advantages of paired electrolysis for efficient utilization of both half‑reactions and the co‑production of two valuable products, while introducing the distinctive capability of generating net electricity. EGCs can be simply defined as spontaneous galvanic cells established by surface electrocatalytic reactions at both electrodes or at a single electrode. In principle, EGCs enable the conversion of renewable biomass, industrial wastes, pollutants, air, or water into commodity chemicals, fuels, or chemical intermediates, concurrently delivering usable electricity (Fig. 1a). This multifunctional nature establishes EGCs as a potentially disruptive complement, or even a sustainable alternative, to conventional fossil fuel-based industrial processes.

**Figure 1.**
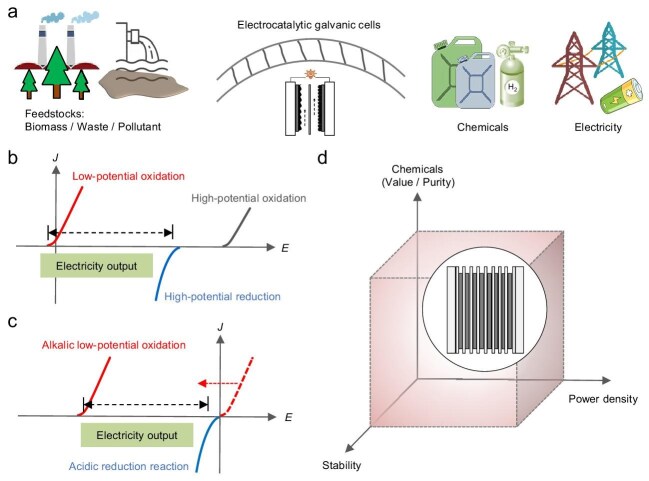
Conceptual framework of EGCs for chemical synthesis and electricity generation. (a) Schematic illustration of EGCs enabling the conversion of renewable biomass, industrial wastes, and pollutants into value-added chemicals with concurrent electricity generation. Two representative strategies for EGCs construction: (b) direct reaction potential pairing; (c) acid-base asymmetric pairing. (d) Vision of scalable EGCs system featuring high power density, high-purity products, and long-term durability.

Two primary strategies have been developed for constructing EGCs. The first and major strategy involves the direct pairing of anodic and cathodic reaction potentials based on their equilibrium potentials (Fig. 1b). The fundamental criterion requires that the equilibrium potential of the cathodic reduction reaction must be sufficiently more positive than that of the anodic oxidation reaction to ensure a positive overall cell potential. In this framework, fuel cells can be regarded as a classical subset of EGCs, typically pairing the oxygen reduction reaction (ORR, *E* ≈ 1.23 V vs. RHE (reversible hydrogen electrode)) with the oxidation of fuels such as H_2_, methanol, or ammonia. However, classical fuel cells often fully oxidize fuels to low‑value products like H_2_O, CO_2_, or N_2_, which limits their chemical versatility. Recent advances have extended this concept toward selective electrocatalytic transformations that preserve or enhance molecular value. A representative example is spontaneous pairing of biomass‑derived aldehyde oxidation with ORR, which transforms electrocatalytic biomass upgrading and H_2_ production from electricity input to electricity output. Studies have shown that hydrogen species generated from C–H bond cleavage of aldehydes on metallic Cu preferentially undergo Tafel recombination rather than a Volmer oxidation at a low onset potential (*E* ≈ 0 V vs. RHE) [1]. This mechanism enabled a unique H_2_ and furoic acid co-generation process at the same anode via low-potential furfural oxidation [2]. And the assembled EGCs based on furfural oxidation paired with four‑electron ORR achieved peak power densities approaching 200 mW cm^−2^. Critically, both anodic and cathodic outputs are value‑added chemicals, underscoring the economic promise of reaction potential pairing. Beyond four‑electron ORR, the two‑electron ORR to H_2_O_2_ (*E* ≈ 0.7 V vs. RHE) provides an alternative cathodic half-reaction with a lower thermodynamic potential but significantly higher product value. H_2_O_2_ serves as an environmentally benign oxidant and a promising liquid energy carrier with an energy density comparable to compressed H_2_. On the anode side, glycerol can also be converted into C3 products with a low oxidation potential (*E* ≈ 0.2 V vs. RHE) [3]. By pairing two‑electron ORR with glycerol oxidation, EGCs have demonstrated the co‑production of H_2_O_2_ and glyceric acid while producing electricity on-site, thereby maximizing overall process economics [4]. Other reduction reactions, such as nitrate reduction to ammonia (*E* ≈ 0.69 V vs. RHE), further expand the suite of viable pairings. When paired with low-potential oxidation reactions such as formaldehyde oxidation (*E* ≈ −0.22 V vs. RHE), such EGCs simultaneously realize wastewater remediation, chemical synthesis and electricity generation [5]. The utilization of industrial pollutants as both electron donors and acceptors highlights a defining strength of EGCs that environmental liabilities can be transformed into chemical and energetic assets.

A second distinct strategy capitalizes on acid-base asymmetry to harness the chemical potential inherent in pH gradients (Fig. 1c). In such acid-base asymmetric EGCs, the anode and cathode operate in spatially separated alkaline and acidic environments, respectively, with an ion-conductive membrane maintaining the pH differential while permitting ionic transport. By strategically pairing pH-dependent electrocatalytic reactions at each electrode, a portion of the acid-base neutralization energy can be converted into electrical energy [6]. The neutralization of one mole of H⁺ with 1 mole of OH^−^ to form water releases ∼79.9 kJ of theoretical energy, corresponding to a theoretical potential difference of 0.828 V. For example, pairing acidic hydrogen evolution reaction (HER, *E* ≈ 0 V vs. RHE) with alkaline formaldehyde oxidation yields a spontaneous cell achieving open‑circuit voltages exceeding 1.1 V and peak power density close to 100 mW cm^−2^ [7]. The attainment of such high voltages, despite the intrinsically low potential of HER, underscores the substantial contribution of the pH gradient to the overall driving force. Similarly, the oxidation of glucose to gluconic acid (*E* ≈ 0.07 V vs. RHE) catalyzed by Au_3_Pt twin nanowires can be paired with acidic HER to yield appreciable electricity while producing valuable platform chemicals [8]. These examples demonstrate that acid-base asymmetric pairing provides a versatile pathway to self‑powered electrosynthesis, particularly for biomass‑derived substrates whose reactivity exhibits strong pH dependence.

Given that anodic reactions are generally thermodynamically demanding and kinetically sluggish, low potential oxidation reactions are also essential for constructing efficient EGCs using both strategies. Direct reaction potential pairing offers conceptual simplicity and avoids the need to maintain pH gradients, but its achievable voltage is fundamentally limited by the intrinsic equilibrium potential difference between the paired reactions. In comparison, acid-base asymmetric pairing provides an additional thermodynamic driving force through the chemical potential difference induced by a pH gradient, thereby enabling more stable cell voltages. However, the direct consumption of commercial acids and alkalis offers limited economic benefits. A more promising approach lies in coupling pollutant conversion with electricity generation during the treatment of industrial acidic and alkaline waste streams. Importantly, these two strategies are fundamentally distinct in their operating principles yet highly complementary, suggesting that their integration will create additional opportunities to build highly efficient EGCs.

Despite these advances, several important challenges remain to be further addressed to realize practical EGCs system for integrated chemical synthesis and electricity generation. A primary consideration is power density, which is determined by both the open-circuit voltage and the achievable current density. Although high voltages have been demonstrated using both pairing strategies, further gains will require the identification of oxidation reactions with even lower onset potentials and reduction reactions with higher equilibrium potentials. For instance, pairing acidic ORR with alkaline alcohol or aldehyde oxidation could theoretically yield cell voltages exceeding 1.8 V. Equally important is the enhancement of current density through catalyst design and system engineering. Unlike conventional electrolyzers, EGCs place greater emphasis on achieving balanced reaction kinetics at both electrodes without external bias, imposing stringent requirements on catalyst symmetry and durability. Well-established electrocatalysis strategies, such as tuning adsorption energetics, accelerating rate-determining steps, constructing hierarchical electrode architectures, and optimizing mass and charge transport, should be systematically adapted for efficient EGCs operation. In parallel, straightforward electrode fabrication that combines operational simplicity with effective tunability, universality, and scalability is essential for translating EGCs system from laboratory to practical technologies [9]. Product value and purity represent another major bottleneck. While many EGCs achieve high selectivity or Faradaic efficiency, the practical utility of the products is often limited by downstream separation and purification costs. A promising direction is the integration of EGCs with cascade or tandem processes, in which the primary products are directly utilized in subsequent reactions without intermediate isolation. Such closed-loop designs could substantially improve overall process efficiency and economic viability. Alternatively, when the primary objective is the conversion of recalcitrant wastes or toxic pollutants, lower-value products may be acceptable if effective environmental remediation is achieved. Currently, EGCs rely on a small subset of oxidation reactions involving aldehydes, alcohols or carbohydrates, and reduction reactions dominated by ORR, nitrate reduction or HER. Expanding this scope to include electrochemical organic transformations of higher molecular complexity, such as C−N or C−C coupling reactions and selective partial oxidations, could significantly augment the chemical value of EGCs output. In parallel, exploring alternative reduction reactions may unlock novel combinations of chemical and energetic outputs. Operational stability, particularly under continuous flow conditions, remains a largely unexplored yet essential frontier. Most existing EGCs operate in batch configurations, and continuous flow processes for long-term operation have yet to be implemented. Anodic catalysts based on Cu or noble metals frequently suffer from deactivation, reconstruction or dissolution, complicating their integration into membrane electrode assemblies. Moreover, membrane degradation, reactants or products crossover, and pH gradient decay pose additional challenges for durable operation [10]. Addressing these issues will require concerted advances in catalyst robustness, membrane materials and reactor engineering.

In summary, EGCs fundamentally redefine the role of electrocatalysis, shifting the paradigm from energy-intensive electrolysis toward simultaneous chemical production and net electricity generation. By unifying feedstocks valorization, green chemical synthesis and electricity generation within a platform, EGCs offer a compelling vision for future sustainable technologies. Continued advances in reaction design, catalyst development and system integration are poised to unlock scalable EGCs with high power density, high-value outputs and robust long-term stability. These developments position EGCs as a versatile and transformative platform for sustainable chemical manufacturing and energy conversion, thereby contributing meaningfully to carbon neutrality and the realization of a circular economy.
